# Evaluation of an enhanced cleaning and disinfection protocol in *Salmonella* contaminated pig holdings in the United Kingdom

**DOI:** 10.1371/journal.pone.0178897

**Published:** 2017-06-08

**Authors:** Francesca Martelli, Mark Lambert, Paul Butt, Tanya Cheney, Fabrizio Antonio Tatone, Rebecca Callaby, André Rabie, Rebecca J. Gosling, Steve Fordon, Graham Crocker, Robert H. Davies, Richard Piers Smith

**Affiliations:** 1 Department of Bacteriology, Animal and Plant Health Agency, Addlestone, United Kingdom; 2 National Wildlife Management Centre, Animal and Plant Health Agency, Sand Hutton, United Kingdom; 3 ADAS, Pendeford, United Kingdom; 4 Department of Epidemiological Sciences, Animal and Plant Health Agency, Addlestone, United Kingdom; 5 Quill Productions, Pulham, United Kingdom; Ross University School of Veterinary Medicine, SAINT KITTS AND NEVIS

## Abstract

*Salmonella* is the second most commonly reported zoonotic gastrointestinal pathogen in the European Union, and a significant proportion of the cases are linked to the consumption of contaminated pork. Reduction of *Salmonella* at the farm level helps to mininimise the contamination pressure at the slaughterhouse, and therefore the number of *Salmonella* bacteria entering the food chain. Cleaning and disinfection (C&D) between batches of pigs is an intervention measure that has potential to reduce the transmission of *Salmonella* contamination within farms. In this study, two pig finisher buildings in each of 10 *Salmonella* positive farms were sampled pre-C&D, post-C&D, post-restocking with the following batch of pigs, and shortly before these pigs were sent to slaughter. The incoming batch of pigs was also sampled before it reached the study building (pre-restocking). At each visit, pooled and individual faecal samples were collected and *Salmonella* isolation was carried out according to an ISO 6579:2002 Annex D-based method. One building on each farm (intervention) was cleaned and disinfected according to a rigorous protocol consisting of several steps and a Defra-approved disinfectant used at the General Orders concentration, whilst the other building (control) was cleaned and disinfected as per normal farm routine. At the post-C&D visit, Enterobacteriaceae and total bacterial counts were determined to evaluate residual faecal contamination and general hygiene levels. Rodent specialists visited the farms before and after C&D and rodent carcasses were collected for *Salmonella* testing. The intervention buildings were significantly less likely (p = 0.004) to be positive for *Salmonella* after C&D. The pre-restocking pigs had the highest likelihood (p<0.001) of being *Salmonella* positive (often with multiple serovars) and there was no significant difference between intervention and control buildings in *Salmonella* prevalence at the post-restocking visit (p = 0.199). However, the pigs housed in the intervention buildings were significantly less likely (p = 0.004) to be positive for *Salmonella* at slaughter age. Multivariable analysis suggested that cleaning all fixtures of buildings, leaving the pens empty for 2–3 days and using an effective disinfectant are factors significantly improving the likelihood of removing *Salmonella* contamination during C&D. Signs of rodents were recorded in all farms, but rodent activity and harbourage availability decreased between visits. All the rats tested were *Salmonella* negative. *S*. Typhimurium or its monophasic variants were isolated from 6 mouse carcasses in 3 farms where the same serovars were isolated from pigs. This study demonstrates that an appropriate C&D programme significantly reduces the likelihood of residual contamination in *Salmonella* positive pig buildings, and suggests a significant reduction in the prevalence of *Salmonella* in the pigs in appropriately cleaned and disinfected buildings when sampled before slaughter. Due to a high prevalence of infection in replacement pigs, control of *Salmonella* in pig farms is challenging. Rodents may also contribute to the carry-over of infection between batches. C&D is a useful measure to help reduce the number of infected pigs going to the slaughterhouse, but should be supplemented by other control measures along the pig breeding and production chain.

## Introduction

*Salmonella* is the second most commonly reported zoonotic gastrointestinal pathogen in the European Union (EU), with 88,715 confirmed human cases reported in 2014 [1]. Although the majority of foodborne outbreaks have been linked to the consumption of eggs and egg products (44.0%), a significant proportion of the outbreaks originate from pork and pork products (9.3%) [1]. In the EU there is no harmonised statutory control for *Salmonella* in live pigs, but pig carcasses are monitored according to the microbiological criteria for foodstuffs defined by Commission Regulation 2073/2005. In the most recent survey conducted on pig farms in the EU in 2008, based on testing of 10 pooled faecal samples per farm, the herd prevalence in nucleus and multiplier farms in the United Kingdom (UK) was 52.2%, and 44.0% in farrow to weaner-grower-finisher farms [2, 3]. In a more recent survey conducted in UK pigs at slaughter, *Salmonella* was isolated from 30.5% of individual caecal content samples and 9.6% of carcass swab samples [4]. Specific slaughterhouse interventions (for example scalding, singeing and blast chilling) can significantly reduce the prevalence of *Salmonella* on pig carcasses, and are more economic and likely to produce larger reductions of human illness than interventions at primary production [5, 6]. Nonetheless, a reduction of *Salmonella* intestinal carriage of live pigs should help reduce the contamination pressure at the slaughterhouse and the environment in pig farming areas that is exposed to pig faecal waste and dust [7, 8]. Biosecurity measures correctly implemented on farm are therefore instrumental in reducing *Salmonella* carriage in live pigs and consequently the number of *Salmonella* contaminated carcasses entering the food chain [9].

Cleaning and disinfection (C&D) of pig pens is considered an essential part of any successful on-farm *Salmonella* control regimen [10, 11]. *Salmonella-*free pigs placed in a contaminated environment are likely to become infected [12] and residual environmental *Salmonella* contamination before placing a new batch of pigs has been shown to increase the risk of *Salmonella* shedding [13]. However, C&D alone is not sufficient to eliminate *Salmonella* contamination from a pig herd [9]. In a model developed to investigate the effectiveness of on farm interventions, it was estimated that the prevalence of *Salmonella* was lower if C&D took place (predicted reduction of 8.0%), but that C&D alone failed to eliminate *Salmonella* from contaminated farms [6]. *Salmonella* has the ability to survive in the environment for several months to years, especially when protected by organic matter such as dried faeces and dust [14, 15]. The effective removal of organic matter is crucial to eliminate *Salmonella* from farm buildings, but this can be hindered by the presence of cracks and crevices in floors, walls and ceilings and the formation of biofilms [16, 17]. The ability of disinfectants to eliminate *Salmonella* is influenced by the type of disinfectant chosen and its concentration, and may be severely compromised by the presence of organic matter [16, 18, 19]. Different types of disinfectant are commercially available, such as quaternary ammonium compounds (QAC) products containing glutaraldehyde or formaldehyde, peroxygen or peracetic acid based compounds, iodine based compounds or chlorocresols. Their effectiveness against *Salmonella* varies greatly, as demonstrated in several *in vitro* and on farm studies [16, 18, 20–23]. Currently, disinfectants intended for veterinary use may be assessed for efficacy using standardised methods of either suspension or surface types, which do not use the matrices commonly found on farms, and therefore the efficacy of a disinfectant in field conditions can be overestimated [22]. In the UK, the Department for Environment, Food and Rural Affairs (Defra) maintains a list of disinfectant products that are approved for use in outbreak situations (http://disinfectants.defra.gov.uk/DisinfectantsExternal/Default.aspx?Module=ApprovalsList_SI). *Salmonella* is reduced by at least 5 logs *in vitro* using Defra General Orders (GO) concentrations of approved disinfectants, but disinfection of faecally contaminated surfaces may be more difficult.

After C&D, bacteriological monitoring can be carried out by assessing the total residual aerobic flora (total bacterial counts, TBC) [24] or by evaluating the residual faecal contamination by isolating the remaining Enterobacteriaceae [25]. Rodents can become infected by *Salmonella* and carry the infection for several months [26–28]. The presence of rodents on a farm can undermine the effectiveness of C&D, as *Salmonella-*carrying mice and rats can re-contaminate cleaned surfaces, in particular feeders and drinkers, and recycle the infection from one batch to the next [10, 29, 30]. The control of rodents on pig farms is of general importance because of their ability to carry several pathogens, and for the economic losses they cause by damaging infrastructure and consuming feed [10].

Within this study, a C&D regimen consisting of disinfectants of known efficacy applied at Defra GO rates and following a rigorous standardised procedure was compared to farmers’ routine C&D procedures on 10 *Salmonella* contaminated pig holdings in the UK. Rodent specialists visited the study farms to assess levels of infestation, sampled rodent populations (by trapping) and provided advice on how to tackle rodent issues. The effectiveness of C&D procedures was evaluated by the reduction in TBC, Enterobacteriaceae and *Salmonella* contamination. The pigs of the batches introduced in the study buildings after C&D were sampled during the rearing period, up to slaughter age, to evaluate the long term effects of the C&D procedures and rodent control on *Salmonella* shedding by pigs close to slaughter.

## Materials and methods

### Holdings selection and sample collection

**Fig 1:**
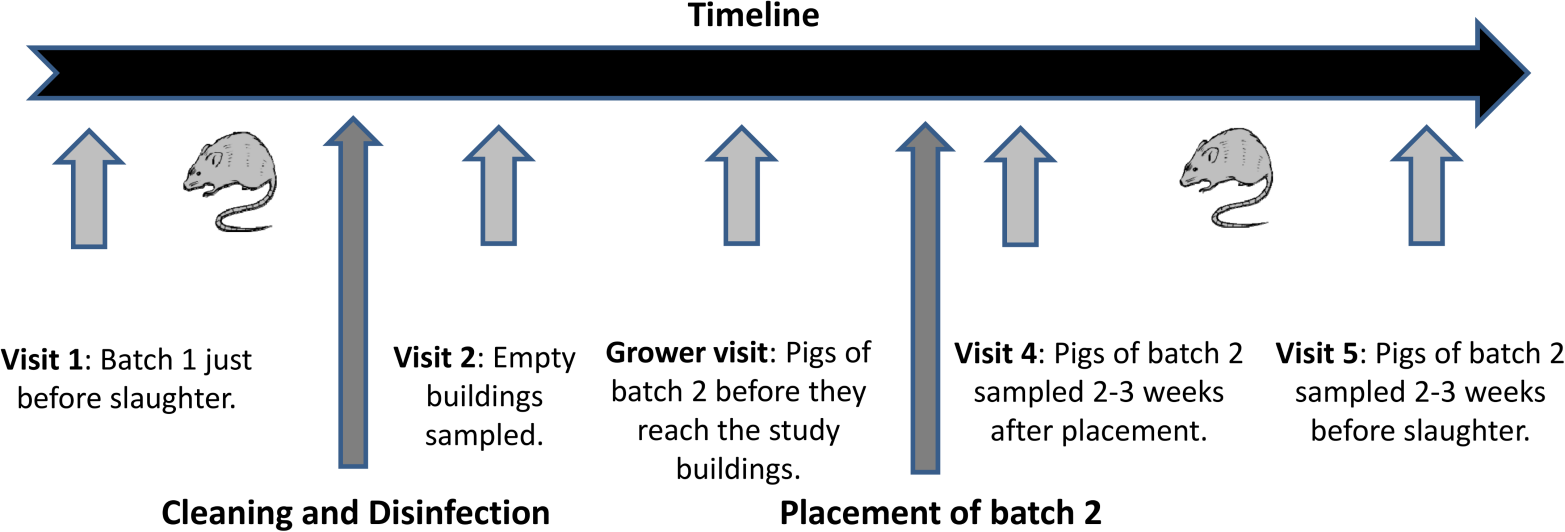
Graphic schematic representation of the timeline of the visits carried out in the 10 study farms.

Ten farms (identified as 221C to 230C) were enrolled in this study (7 wean to finish, 2 grow to finish and 1 farrow to finish). All farms were known to be positive for *Salmonella*, on the basis of results from scanning surveillance or previous investigations (data not shown). All farms produced finished bacon pigs, operated the study buildings using an all-in/all-out programme, and had a previous pen faecal prevalence of *Salmonella* of over 20%. In each of the 10 farms, two buildings were selected. These were buildings housing finishing pigs at the same stage, with similar size and management practices (e.g. same feeder and drinkers types, same intercrop routine, same source of pigs). One building was randomly selected as the intervention building, and the other building served as control. Four sampling visits were carried out within this trial to each of the study farms, over the life of two batches of pigs. The first visit (pre-C&D) was carried out when the first batch of pigs was close to slaughter (at least 2 weeks before the buildings were empty, and when the majority of pigs were still housed in the study buildings). The second visit (post-C&D) was carried out when the buildings had been cleaned and disinfected and were still empty. The third visit was carried out 2 to 3 weeks after the second batch of pigs had been housed in the study buildings (post-restocking). The fourth visit was carried out when the second batch of pigs was close (2 to 3 weeks) to slaughter (pre-slaughter), as for visit one. Additionally, for the 7 wean to finish farms, the pigs of the second batch were sampled at the breeding site of origin, to gather baseline data of the *Salmonella* prevalence in the batch before the pigs were placed in the study buildings (pre-restocking). A schematic representation of the timeline of the farm visits is provided in Fig 1. For the remaining 3 farms, such sampling was carried out in the weaner buildings present on farm during the second sampling visit. When sampling occupied buildings, a pen level sampling strategy was adopted.

At each visit, a questionnaire was completed by the participating farmer, to capture farm management data. In particular information on the C&D routine (e.g. how long the pens were left empty between batches, details of the application of the farm C&D regimen) was collected. The questionnaire was administered by a member of the study team at each visit, and served the purpose of gathering information on the management of the farm in a structured way. No views of the individuals involved or any personal characteristics were surveyed. All farmers signed a written consent to participate in the study. The administered questionnaire was not reviewed by a Review board before the start of the study, as the number of participants was limited (10) and only information on farm management was collected. Defra’s policy is to submit to a survey control unit any questionnaire if the number of respondents is larger than 25.

One pooled faecal sample was collected for each 50 pigs housed in a pen. Furthermore, 10 individual faecal samples were collected from the floor in up to 6 randomly selected pens for each building. At the second visit (post-C&D), feeders, drinkers and floors of the empty buildings were swabbed with hand-held gauze swabs. Up to ten randomly selected pens (or all available pens if less than 10) were sampled in each building at this visit. With each swab, 0.5 m^2^ of surface was sampled by thorough swabbing back and forth in both horizontal and vertical directions.

### Cleaning and disinfection procedures

All intervention buildings were cleaned and disinfected by trained contractors according to a standardised protocol, comprising a series of steps (removal of faeces, foaming, washing, disinfecting and cleaning portable equipment). Firstly all portable equipment was removed, the building dry cleaned and the water lines emptied and refilled with 2% Intra Hydracare (Intracare, Veghel, The Netherlands) solution. Secondly the building was pressure washed and Target Powergel (Evans Vanodine International Plc, Preston, UK) (5%) applied with a foaming lance. At this stage the drinking system was emptied again and flushed with clean water. In the third stage, debris and foam were removed by pressure washing, and the building was left to dry for 24 hours. When dry, GPC8 (Evans Vanodine International Plc, Preston, UK) at Defra GO concentration (1:35 parts of disinfectant to parts of water) was applied (either by power wash or foaming) and boot dips were refilled with FAM30 1:90 (Evans Vanodine International Plc, Preston, UK). The 1:35 GPC8 dilution equates to a concentration of 2.957% w/w (0.3767% w/w glutaraldehyde and 0.14315 w/w didecyldimethylammonium chloride). GPC8 was applied on surfaces to saturation (run-off) point, corresponding to approximately 6 L of diluted product per square meter. At this point all portable equipment was disinfected by spray with GPC8 1:35. At the last stage, the cleaned and disinfected equipment was replaced in the buildings and the water lines were refilled with clean water.

The procedures and products used in the control buildings were those usually employed by the farmer, and therefore differed from farm to farm. The farmer C&D practices were recorded by each farmer in a questionnaire. In all farms the residual faeces and straw were removed from the buildings and the floors power washed with cold water before the start of the disinfection. Table 1 summarises the types of disinfectant used in the control buildings in each farm and their dilution rates.

**Table 1:** Types of disinfectants used in the control buildings in each farm and their concentration in relation to the Defra General Orders concentration of that product.

Farm	Disinfectant class	GO dilution rate ^a^	Dilution rate on farm ^a^	Water lines and water tank disinfected?
221C	Iodide	1:90	1:50	Yes
222C	Glutaraldehyde and QAC	1:33	1:200	Yes
223C	Iodide	1:50	1:100	No
224C	Glutaraldehyde and QAC	1:35	1:49	Not known
225C	Potassium peroxymonosulfate	1:100 ^b^	1:100 ^b^	No
226C	Potassium peroxymonosulfate	1:100 ^b^	1:100 ^b^	No
227C	Potassium peroxymonosulfate	1:100 ^b^	1:100 ^b^	No
228C	Glutaraldehyde and QAC	1:33	1:200	Yes
229C	Glutaraldehyde and QAC	1:33	1:200	Yes
230C	Glutaraldehyde and QAC	1:33	1:200	Yes

^**a**^ One Part Disinfectant to Parts of Water, unless otherwise indicated.

^b^ 1 g of disinfectant in 100ml of water

### Sample testing

Approximately 25g of pooled pen faeces samples were collected with sterile hand held gauze swabs and then placed directly into 225ml of the pre-enrichment culture media (Buffered Peptone Water–BPW;Merck 10.07228.0500) at the farm. Ten grams of individual faeces were returned to the laboratory, weighed out and added to 90ml of BPW. All BPW samples were incubated at 37±1^°^C for 16–20 hours and subsequently 0.1ml of broth was inoculated onto modified semi-solid Rappaport-Vassiliadis (MSRV; Mast DM440D, with addition of 1mg/ml of novobiocin, Sigma N1628) enrichment agar and incubated at 41.5±1^°^C for 24 ± 3 hours. Growth on MSRV was sub-cultured onto Rambach agar (Merck 1.07500.0002) which was incubated at 37±1^°^C for 24 ± 3 hours. Slide agglutination tests on suspect colonies from samples were also carried out to confirm *Salmonella* positive results. The positive samples were subjected to serotyping [31]. At the post C&D visit, the swab was introduced into 225ml of BPW on farm and then shaken vigorously. A 1ml aliquot of the BPW was taken and diluted tenfold 3 times. All dilutions were plated on farm onto Violet Red Bile Glucose Agar (VRBG) agar and Sheep Blood Agar (SBA) to estimate Enterobacteriaceae and total aerobic bacteria, respectively. The remaining BPW samples and the plates were then incubated at 37±1^°^C for 16–20 hours. Colonies on VRBG and SBA plates were then counted and if no growth was observed, 0.1 of incubated BPW was plated on SBA and VRBG. *Salmonella* isolation was carried out as described above.

### Rodents

A search of the farm buildings and surrounding areas was carried out; signs of rodent activity were recorded on a plan of the site (generated using Ordnance Survey® map data) onto which a 25m x 25m grid was superimposed in ArcMapTM Version 10.2 (Esri®, California). The rodent activity signs, including rodent runs, burrows, droppings, urine pillars, disturbed bait and smears [32] were recorded as either recent or non-recent; rat runs for instance were recorded as fresh if they were smooth, well-trodden and had no signs of recent plant growth, burrows were recorded as fresh if they had no cobwebs or obstructions at the entrance or there were recent signs of digging.

The level of harbourage (potential rodent nesting and breeding areas) within 20m of the farm buildings was subjectively assessed as absent, low, medium, high or very high (these were given scores of 0–4 respectively).

Automated trail cameras (Reconyx Hyperfire^TM^, Reconyx Inc., Wisconsin, USA) were used in areas where recent evidence of rodent activity was seen (and potential rodent habitats) to determine the level of rodent activity with a maximum of one camera in each 25m x 25m grid square. A General Index (GI) of activity [33] was calculated for Norway rats and house mice as the average number of images per camera per night. In a previous study, data from camera traps were compared with results from established census methods to enable conversion of activity indices from camera data to population estimates for Norway rats, but we were unable to validate activity indices for house mice [34]. To explore the relationship between activity indices (from cameras) and population size for house mice from trapping data, here we examined the change in the activity index from camera data following removal of a known number of house mice from site 224C. The change in activity index divided by the number of mice removed from grid squares with cameras gave an upper estimate for the contribution to the activity index per mouse; the change in activity index divided by the total number of mice removed gave a lower estimate.

Following removal of the trail cameras, traps were set to obtain a sample of rats and mice (where present) for the isolation of *Salmonella*. We aimed to obtain a sample size of 11 Norway rats and 11 house mice where possible in order to be able to detect *Salmonella* prevalence of 25% or greater within the population with 95% confidence. Traps were set according to signs of recent activity; where sufficient signs of activity were present the number of trap nights was at least twice the number of samples required. Any live rodents captured were humanely killed and transported on ice to the laboratory for *Salmonella* isolation. The exterior of the rodent carcasses was disinfected with a potassium peroxymonosulphate product before they were cut open with a sterile scalpel. The intestine, liver, kidneys and spleen were aseptically removed, added to 225ml of BPW and *Salmonella* isolation was performed as described above.

The rodent survey and sampling were repeated for each site following depopulation, cleaning, disinfection and re-stocking of the buildings. A paired T test was used to compare the harbourage scores and rodents activity indices between baseline and follow up visits.

### Statistical analysis

The association between intervention type (C&D with either the intervention or control method) and the shedding of *Salmonella* by pigs on the farms was assessed using generalised linear mixed models (GLMM) fitted with a logit link function and binomial errors and a Laplace approximation to the maximum likelihood estimation in *R version 3*.*2*.*4* using the *lme4* package [35]. All models had the following model structure:

$$\begin{array}{l} \mathrm{logit}(Salmonella_{i})=\\ \alpha +\beta_{1}\mathrm{Sample}\;\mathrm{Type}+\beta_{2}\mathrm{Season}+\beta_{3}Intervention*\beta_{2}\mathrm{Visit}\;\mathrm{Type}+b_{1_{i}}\mathrm{Farm}\;{\mathrm{ID}}_{i}+\varepsilon_{i} \end{array}$$

where *α* is the intercept. The outcome variable *Salmonella* is the binary presence/absence of *Salmonella* in a sample. Sample type (whether a pooled sample or not) and season of sampling (a 4 level categorical variable with spring as the reference group, as this season had the most samples taken) were included in the model as *a priori* fixed effect variables (β). An interaction term was fitted between the visit type (categorical variable) and intervention type (intervention or control) to assess the effect of the intervention over time. Farm identity (FarmID) was included in the model as a random effect (b).

Using the above model structure, three GLMMs were built to compare intervention and control results from:

1. The pre-C&D and post-C&D visits
2. The pre-C&D and post-restocking visits
3. The pre-C&D and pre-slaughter visits to the farm

For points 2 and 3, the *Salmonella* prevalence of the pigs at the pre-restocking visit was included as a fixed effect in the model.

As the control buildings were cleaned using different methods and changes occurred on the farms which may have explained changes in results between visits, this GLMM was further explored. A forwards stepwise selection process was used to identify variables collected from the farm questionnaires that were significantly associated with *Salmonella* prevalence to evaluate whether specific differences between the farms and their cleaning methods influenced the outcome of this trial. Sample type and age group of pigs sampled, season and whether the sample came from an intervention building or a control, were included in the model as *a priori* variables, and data from the pre-restocking visit were excluded from the model. The variables included in the model are detailed in Table 2.

**Table 2:** Multivariable analysis of factors identified as associated with *Salmonella* in the 10 study farms at all visits except the pre-restocking visit (significant values in bold). When the levels of a variable were collinear with the effect of a different value, no Odd Ratio (OR) values are produced by the model.

Variable	Level	No. positive	No. samples	% positive	OR	P value	95% CI	
Sample type	Individual	481	2,904	16.6	1.00			
	Pooled	471	1,741	27.1	4.48	**<0.001**	3.68	5.45
Season	Winter	94	596	15.8	1.00			
	Spring	325	1,740	18.7	1.10	0.856	0.40	3.05
	Summer	308	953	32.3	1.54	0.331	0.64	3.68
	Autumn	225	1,356	16.6	2.89	**0.002**	1.46	5.74
Intervention building	No	465	2,298	20.2	1.00			
	Yes	487	2,347	20.7	0.93	0.872	0.39	2.24
Pig age group sampled	Farrowing	36	115	31.3	1.00			
	Weaners	182	478	38.1	43.34	**0.002**	3.92	479.08
	Growers	65	511	12.7	0.05	**0.049**	0.00	0.99
	Finishers	645	2,873	22.5	0.15	0.151	0.01	2.01
	Not Applicable	24	668	3.6	0.15	0.046	0.02	0.97
How long are Pens left empty	1–2 days	165	756	21.8	1.00			
	3–4 days	22	441	5.0	0.02	**<0.001**	0.00	0.16
	7–10 days	182	667	27.3	0.09	**<0.001**	0.02	0.34
	2 weeks	232	915	25.4	10.51	**0.009**	1.81	60.93
	2–3 weeks	82	736	11.1	5.18	**0.035**	1.12	23.90
	Missing	231	746	31.0	0.56	0.257	0.21	1.52
	Not known	38	384	9.9	20.74	0.009	2.11	203.40
Building areas cleaned	Vents, beams, Ceiling, Ledges	279	1,858	15.0	1.00			
	Beams, Ceiling, Ledges	25	439	5.7	0.00	**<0.001**	0.00	0.04
	Ledges only	371	977	38.0	9.60	**0.001**	2.38	38.64
	Missing	120	933	12.9	0.05	0.037	0.00	0.83
	Vents, beams, Ledges	157	438	35.8	0.02	**<0.001**	0.00	0.08
Treatments used since last visit	0	514	2,255	22.8	1.00			
	1	58	504	11.5	0.23	**<0.001**	0.10	0.50
	2	52	414	12.6	0.63	0.301	0.27	1.51
	10	4	140	2.9	0.23	**0.036**	0.06	0.91
	Baseline	324	1,332	24.3	0.42	0.099	0.15	1.18
C&D disinfectant used	GPC8	498	2,565	19.4	1.00			
	Iodine product 1	106	250	42.4	3.21	**0.022**	1.18	8.74
	Iodine product 2	63	162	38.9	0.55	0.436	0.13	2.44
	Potassium peroxymonosulfate 1	11	252	4.4	0.54	0.401	0.13	2.26
	Potassium peroxymonosulfate 2	105	398	26.4	0.20	**0.029**	0.05	0.84
	Glutaraldehyde and QAC	169	1,018	16.6	0.17	**0.010**	0.05	0.66
Any medicine used in that group up to 12 months before 1st visit	No	255	2,112	12.1	1.00			
	Yes	697	2,533	27.5	7.35	**<0.001**	3.09	17.46
Bedding type used by group	None	226	1,639	13.8	1.00			
	Other	17	44	38.6	1.62	0.441	0.48	5.48
	Straw	709	2,962	23.9	0.23	**<0.001**	0.13	0.42
Ventilation system	Roof vent	64	635	10.1	1.00			
	Side vent	455	2,140	21.3	6.96	**<0.001**	2.37	20.48
	Not Applicable	433	1,870	23.2	6.38	<0.001	2.71	14.98
Building cleanliness score	2 (poor)	61	263	23.2	1.00			
	3	233	1,095	21.3	0.06	**<0.001**	0.02	0.19
	4	434	2,103	20.6	0.15	**0.001**	0.05	0.44
	5 (excellent)	128	332	38.6	0.10	0.057	0.01	1.07
	Missing	96	852	11.3	0.07	<0.001	0.02	0.28
Feeding change between visits	Baseline ^a^	324	1,332	24.3	1.00			
	Change	7	144	4.9	20.06	**0.001**	3.59	112.11
	No-change	621	3,169	19.6	-			
Time left to dry before repopulating	1–2 days	509	2,817	18.1	1.00			
	3–4 days	332	1,283	25.9	4.50	**0.005**	1.58	12.80
	5 days	106	250	42.4	-			
	7+ days	5	295	1.7	-			
Coughing in group at visit	No	917	3,872	23.7	1.00			
	Yes	11	105	10.5	33.00	**0.015**	1.96	555.19
	Not Applicable	24	668	3.6	-			
Wildlife situation change between visits	Baseline ^b^	324	1,332	24.3	1.00			
	Better	42	284	14.8	0.17	**0.019**	0.04	0.75
	No-change	572	2,958	19.3	0.43	0.088	0.17	1.13
	Worse	14	71	19.7	-			

^**a**^ Information as collected at the pre cleaning and disinfection visit.

^b^ Assessment of wildlife at the pre cleaning and disinfection visit.

When analysing the Enterobacteriaceae and total bacterial counts, statistical analyses were carried out with STATA® software (StataCorp, Texas, USA). An arithmetic mean and standard deviation were obtained for TBC and Enterobacteriaceae counts from each farm. The bacterial counts were converted into log_10_ colony-forming units per 50cm^2^. Then, histograms of the data obtained were created to characterize the distribution of the variables. In order to test for statistical significance between the intervention and control results, it was decided to use a negative binomial model. The model tested both Enterobacteriaceae and TBC counts as two separate outcomes, and included the farm identifier as a random effect to account for the non-independence of samples from the same farm. The fixed effects included whether the samples came from the intervention or control building and the location of the samples (i.e. floor, feeder and drinker), whilst also accounting for seasonality by including month and season when the samples were collected. The season variable was omitted from the final models due to collinearity with month. Differences with p value<0.05 were considered statistically significant.

## Results

### *Salmonella* prevalence and serovars

The number of *Salmonella* positive samples at each visit in each of the study farms, including *Salmonella* serotyping results, is detailed in Table 3. *Salmonella* was isolated from all control and intervention buildings at the pre C&D visit, apart from the control buildings in farms 222C and 225C. *S*. Typhimurium and/or its monophasic variants (*S*. 4,[5],12:i:-) were isolated from all farms at the first visit. At the post C&D visit, *Salmonella* was isolated only in farm 228C in the intervention building, and in farms 221C, 229C and 230C in the control buildings. All the groups of pigs sampled at the pre-restocking visit (apart from those supplied to farm 222C) tested positive for *Salmonella*, and in 5 batches multiple serovars were isolated. At the post- restocking visit, *Salmonella* was isolated from all buildings in all farms, except for the intervention building in farm 225C. At the pre-slaughter visit, *Salmonella* was isolated from all buildings in all farms, except for farm 222C where *Salmonella* was not isolated at this visit.

**Table 3:** *Salmonella* isolated from the 10 study farms at the 4 sampling visits in pooled and individual faecal samples (PF and IF, respectively) and in floors (Fl), feeders (Fe) and drinkers (Dr) at the post-C&D visit (number of *Salmonella* positive samples/number tested). Positive at the post-C&D visit are in bold. Results of testing of faeces of the pigs of batch 2 before they reached the destination study buildings are also reported in the table.

	Pre-C&D				Post-C&D						Pre-restock	Post-restocking				Pre-slaughter			
	Intervention		Control		Intervention			Control				Intervention		Control		Intervention		Control	
	PF	IF	PF	IF	Fl	Fe	Dr	Fl	Fe	Dr	PF and IF	PF	IF	PF	IF	PF	IF	PF	IF
**221C**	1/28^b^	0/30	8/28^a^^,^^b^^,^^c^	9/30^a^	0/18	0/6	0/6	**6/18**^**d**^	0/6	**1/6**^**n**^	59/161 ^a^^,^^**d**^^,^^g^	18/18^a^^,^^**d**^	27/60^a^^,^^**d**^	16/20^a^^,^^**d**^	47/60^a^^,^^**d**^	5/18^a^^,^^e^	4/60^a^	3/18^a^^,^^**d**^	15/60^a^^,^^e^
**222C**	6/20^b^	5/30^b^	0/20	0/30	0/10	0/10	0/10	0/10	0/10	0/10	0/65	6/20^a^^,^^b^	6/60^a^^,^^b^	1/20^a^	0/60	0/20	0/60	0/20	0/60
**223C**	1/15^b^	3/30^b^	10/15^b^	11/29^b^	0/19	0/8	0/2	0/27	0/1	0/2	52/196 ^b^^,^^g^^,^^**d**^	14/15^a^	26/30^a^	13/15^a^	11/30^a^^,^^b^	7/15^a^^,^^b^	2/28^a^^,^^b^	11/15^a^^,^^b^	4/28^a^^,^^b^
**224C**	4/14^e^	0/30	2/9^e^^,^^b^	0/30	0/10	0/10	0/10	0/10	0/10	0/10	41/112^a^	1/16^e^	3/59^e^^,^^f^	1/14^e^	4/59^e^	2/15^f^	1/58^f^	2/7^a^^,^^e^	2/60^a^^,^^e^
**225C**	2/12^a^	1/21^a^	0/24	0/30	0/10	0/10	0/10	0/10	0/10	0/10	2/140^e^	0/11	0/60	2/11^a^	0/59	1/12^a^	1/49^a^	3/12^a^	0/57
**226C**	18/19^e^^,^^g^	17/30^a^^,^^e^^,^^g^	5/12^e^	11/30^a^	0/10	0/10	0/10	0/10	0/10	0/10	69/240^e^	17/20^a^^,^^**d**^^,^^e^^,^^g^^,^^h^	21/60^a^^,^^e^^,^^g^	19/20^e^^,^^g^	20/30^e^	10/12^e^^,^^h^	11/60^e^^,^^h^	5/12^a^^,^^e^	9/60^e^
**227C**	9/12^e^	2/10^e^	6/12^e^	7/30^e^	0/10	0/10	0/10	0/20	0/20	0/20	69/240^e^	11/12^e^	17/60^e^	4/12^e^	3/60^e^	5/12^e^	9/60^e^	6/12^e^^,^^g^	9/60^e^
**228C**	8/28^a^	10/29^a^	12/28^a^	6/30^a^	**1/10**^a^	**1/10**^a^	0/10	0/10	0/10	1/10	37/118 ^a^^,^^b^^,^^h^^,I,m^	14/14^a^^,^^h^^,^^i^	32/59^a^^,^^e^	5/14^a^	8/60^a^^,^^i^	6/28^a^	6/60^a^	20/28^a^^,^^i^	15/60^a^^,^^e^
**229C**	27/28^e^	22/30^e^	25/28^e^	10/30^e^	0/10	0/10	0/10	**2/10**^e^	**1/10**^e^	0/10	20/117 ^a^^,^^**d**^^,^^h^^,^^g^^,I,m^	8/26^a^	2/60^a^	6/28^a^^,^^g^	4/60^a^^,^^g^	6/28^a^	1/60^a^	13/28^a^^,^^f^	11/60^a^
**230C**	5/10^a^^,^^b^	6/30^b^	6/10^b^	0/30	0/10	0/10	0/5	**3/9**^a^	0/9	0/4	66/84^a^^,^^i^	5/10^a^^,^^i^	16/60^a^^,^^h^^,^^i^	5/10^a^^,^^i^	9/60^a^^,^^i^	1/9^i^	0/60	1/10^i^	2/60^a^

^**a**^ *S*. 4,5,12:i:-

^**b**^ *S*. Typhimurium

^**c**^ *S*. Agama

^**d**^ *S*. Reading

^**e**^ *S*. 4,12:i:-

^**f**^ *S*. Rissen

^**g**^ *S*. Bovismorbificans

^**h**^ *S*. London

^**i**^ *S*. Derby

^**l**^ *S*. Bardo

^**m**^ *S*. Newport

^n^ Serotype not determined

Eleven *Salmonella* serovars were identified across the 10 study farms, with a minimum of 1 serovar isolated from farm 225C (*S*. 4,5,12:i:-) to a maximum of 6 serovars isolated from the breeding site supplying farm 229C (*S*. 4,5,12:i:-, *S*. 4,12:i:-, *S*. Bovismorbificans, *S*. Bardo, *S*. London and *S*. Reading). At the pre and post restocking visits, a larger variety of serovars was isolated. The serovars isolated at the post C&D visit in the 4 farms that tested positive was isolated in the same farms at the post restocking visit in all farms but one (229C).

### Statistical analysis

**Fig 2:**
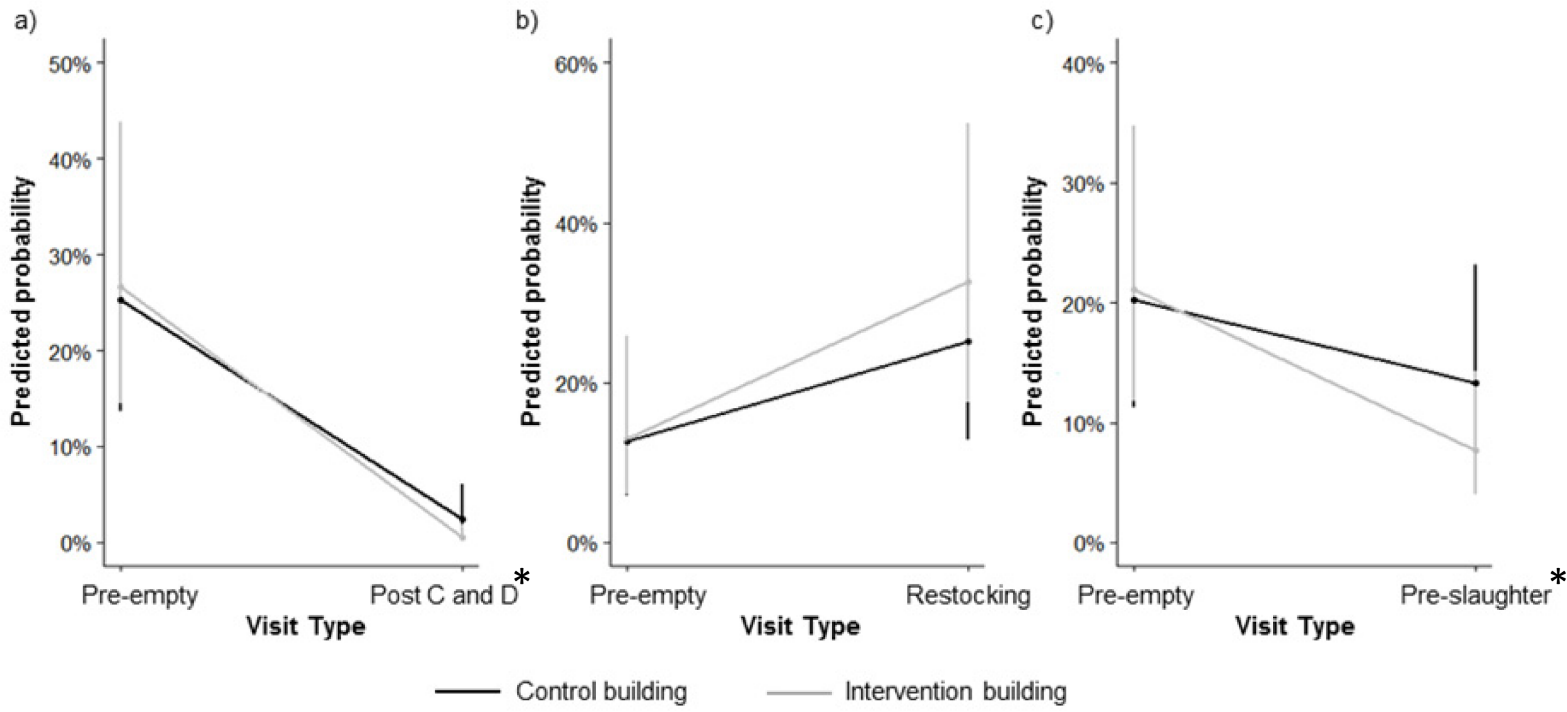
Plot showing the interaction effect of intervention and visit type on the predicted probability of a sample being positive for *Salmonella*. The error bars are the 95% confidence interval for the predicted probability. Samples used in this analysis were: a) from intervention and control buildings at the pre-C&D and post-C&D visits only; b) from intervention and control buildings at the pre-C&D and post-restocking visits only; c) from intervention and control buildings at the pre- C&D and pre-slaughter visits only. An asterisk indicates at which visit significant differences were observed between intervention and control buildings.

At the post C&D visit, the intervention buildings were less likely to be positive for *Salmonella* than the control buildings (p = 0.004) (Fig 2A). The pigs sampled at the pre-restocking visit were more likely to be *Salmonella* positive than the pigs sampled in the intervention and control buildings at the pre-slaughter visits (p<0.001). Taking the prevalence of the grower pigs sampled into account, there was no difference in the likelihood of the intervention and control buildings being positive for *Salmonella* at the post-restocking visit (p = 0.119) (Fig 2B), but the pigs housed in the intervention building were less likely to be *Salmonella* positive at the pre-slaughter visit than the pigs housed in the control buildings (p = 0.004) (Fig 2C). Pooled faecal samples were more likely to be positive for *Salmonella* than individual samples (p<0.001). Samples collected during the summer were more likely to be positive for *Salmonella* than samples collected in spring (p<0.001) and samples collected in winter were less likely to be positive than spring samples (p = 0.005).

The results of the multivariable analysis are detailed in Table 2. A total of 12 significant variables entered the final model, with eight more joining the original four *a priori* variables. The addition of these variables appeared to explain the differences between intervention and control building results, with the intervention variable becoming non-significant. The specific differences in cleaning methods, that were detected as significant, showed that cleaning only ledges but not beams, ceilings and vents was identified as a practice that significantly increased the risk of residual *Salmonella* contamination (p = 0.001; OR 9.60). Also, the use of an iodine-based disinfectant was significantly less likely to remove contamination when compared to GPC8 (p = 0.022; OR 3.21). Leaving pens empty for 3 to 4 days (p<0.001; OR = 0.02) or 7–10 days (p<0.001; OR = 0.09) significantly reduced the likelihood of *Salmonella* contamination when compared to leaving the pens empty for only 1–2 days.

### Enterobacteriaceae and TBC

Table 4 summarises the average counts for all sample types in intervention and control buildings of nine of the study farms (farm 226C was removed as the data available were incomplete). The numbers of Enterobacteriaceae were lower in intervention buildings than in control buildings in five farms (223-C, 227-C, 221-C, 222-C and 229-C). There was no reduction in the remaining farms. Overall, the average Enterobacteriaceae counts were higher in intervention buildings, with a difference of 0.47 log_10_ CFU/50 cm^2^ (p = 0.012). For TBC, there was an average difference between the intervention and the control buildings of 0.16 log_10_ CFU/50 cm^2^, with the control buildings having significantly higher counts (p = 0.018). Table 5 presents the average counts of all farms combined for floors, feeders and drinkers. TBC were statistically significantly lower in intervention buildings in samples from floors, drinkers and feeders (p<0.001) and only in drinker samples (p = 0.003) for Enterobacteriaceae.

**Table 4:** Average Enterobacteriaceae and total bacterial counts (B) in intervention (I) and control (C) buildings of 9 study farms. In the intervention buildings in farms 223C and 227C no Enterobacteriaceae were isolated (N/A).

		Enterobacteriaceae (log10 CFU/50 cm^2^)	Total Bacterial Counts (log10 CFU/50 cm^2^)
221C	I	4.93	6.14
	C	5.27	7.40
222C	I	3.52	6.55
	C	4.27	6.28
223C	I	N/A	8.05
	C	3.60	8.27
224C	I	6.08	7.32
	C	5.11	6.81
225C	I	3.50	5.00
	C	3.45	6.85
227C	I	N/A	6.72
	C	3.96	7.43
228C	I	5.32	7.30
	C	5.10	6.55
229C	I	3.44	5.35
	C	4.82	6.09
230C	I	4.48	7.04
	C	4.29	6.27

**Table 5:** Average Enterobacteriaceae and total bacterial counts in intervention (I) and control buildings (C) in floors, feeders and drinkers of all study farms combined.

		Enterobacteriaceae (log10 CFU/50 cm^2^)	Total bacterial counts (log10 CFU/50 cm^2^)
Floor	I	5.69	7.20
	C	4.94	7.73
Feeders	I	4.01	7.57
	C	4.79	8.67
Drinkers	I	2.85	5.94
	C	4.00	6.79

### Rodents

Rodent presence was detected by cameras or traps at all 10 study farms (Table 6). The availability of harbourage was estimated to be within 0 (none) and 2.5 (average-high). In all farms apart from 227C, the harbourage availability remained the same or decreased between the baseline and follow-up visit. Due to the limited number of farms included in this study, the reduction in harbourage availability was not statistically significant (p>0.05). Rats were recorded by cameras at 6 farms, with activity indices ranging from 1.75 to 326.10 (population estimates between 7 and 501 rats per site). A reduction in Norway rat activity was recorded at 4 farms between the baseline and follow up visits. A total of 39 carcasses of rats were obtained and all tested negative for *Salmonella*. House mice were recorded by cameras at 8 farms, with activity indices ranging from 0.75 and 206.25 (population estimates ranges between 0–1 and 125–181 mice per site). A reduction in the house mouse activity index was observed at 7 farms between the baseline and follow up visit. No data were available for the baseline visit at farm 227C. Due to the limited number of farms included in this study, the reduction in activity indexes for mice and rats was not statistically significant (p>0.05). A total of 85 mice carcasses were obtained and 6 tested positive for *Salmonella*. The carcasses originated from farms 224C, 228C and 230C. At farm 224C a total of 3 carcasses tested positive for *Salmonella* (*S*. Typhimurium (x1) and *S*. 4,12:i:-). *S*. 4,5,12:i:- was isolated from one carcass obtained from 230C and two carcasses from 228C. The *Salmonella* serovars isolated from the mice carcasses were circulating in the pigs in farms 224C, 228C and 230C. Immediately after the second trapping session at site 224C (where 16 mice were captured), the six cameras inside buildings were re-set (in the same positions as for the follow-up survey). Following trapping the house mouse activity index for the site declined to 20.92 (compared to 39.13 for pre-trapping), a reduction of 18.21. Of the 16 mice removed, 11 were from grid squares where cameras were located. Dividing the change in activity index by the number of mice removed from grid squares with cameras (18.21/11) gave a possible conversion factor (i.e. the contribution to the activity index per mouse) of 1.66; dividing the change in activity index by the total number of mice removed (18.21/16) gave an alternative conversion factor of 1.14. These conversion factors were used to obtain a lower and upper estimate of the number of mice in each site where activity indices were available (Table 6).

**Table 6:** Results of rodent surveys per farm (harbourage availability, population estimates, number of carcasses obtained and *Salmonella* testing results). Data are reported for the baseline (B) and follow up visit (F).

	Harbourage availability ^a^		Norway rat activity index (mean number of photographs by camera by night		Population estimate (number of rats for the area surveyed) for Norway rats (range of estimates in brackets)		Rats obtained for testing ^b^^,^ ^c^		House mouse activity index ^d^		Range of population estimates (number of mice for the area surveyed) for house mice ^d^		Mice obtained for testing ^b^^,^^c^	
	B*	F**	B	F	B	F	B	F	B	F	B	F	B	F
**221C**	1	0	4.51	0.00	15 (12–19)	0 (0–0)	1 (0)	0	21.25	0.00	13–19	0–0	0	0
**222C**	2	1	119.25	34.25	221 (173–281)	79 (62–100)	7 (0)	8 (0)	206.25	16.21	125–181	10–14	6 (0)	5 (0)
**223C**	2	1	0.00	0.00	0 (0–0)	0 (0–0)	0	0	9.79	0.75	6–9	0–1	0	3 (0)
**224C**	1	1	0.00	2.10	0 (0–0)	8 (6–10)	1 (0)	0	60.38	39.13	36–53	24–34	**32 (6.25)**	**16 (6.25)**
**225C**	1	1	326.10	317.40	501 (394–638)	490 (385–624)	9 (0)	13 (0)	0.00	0.00	0 (0–0)	0 (0–0)	0	0
**226C**	1	1	0.06	1.75	0 (0–1)	7 (6–9)	0	0	0.75	0.00	0–1	0–0	4 (0)	1 (0)
**227C**	1	2	0.00	0.00	0 (0–0)	0 (0–0)	0	0	No data	79.17	No data	48–70	4 (0)	0
**228C**	1.5	1	10.67	0.00	31 (24–39)	0 (0–0)	0	0	19.44	0.00	12–17	0–0	**2 (100)**	0
**229C**	2.5	2	0.00	0.00	0 (0–0)	0 (0–0)	0	0	0.00	0.00	0–0	0–0	1 (0)	0
**230C**	1	1	0.00	0.00	0 (0–0)	0 (0–0)	0	0	157.75	54.92	95–139	33–48	8 (0)	**3 (33.3)**

* Baseline visit: visit carried out approximately at the time of cleaning and disinfection in the pig buildings

** Follow up visit: visit carrie out during the life of the second batch of pigs placed in the study buildings.

^a^ 0 = none; 1 = low; 1.5 = low-average; 2 = average; 2.5 = average-high

^b^ Includes mice and rats found dead or caught by means other than trapping.

^c^ Percentage of *Salmonella* positive carcasses in brackets. Positive carcasses are in bold.

^d^ From indoor cameras (as house mice are rarely found outside buildings).

## Discussion and conclusions

This study investigated the effectiveness of a standardised C&D protocol when compared to the normal C&D routines carried out in finisher buildings at 10 pig farms in the UK. The product used for disinfection in the intervention buildings (GPC8, Evans Vanodine International Plc, Preston, UK) is glutaraldehyde and QAC based. Aldehyde-based disinfectants have been reported to be more effective than other types in poultry houses [20, 23, 36]. Formaldehyde, particularly if applied by spraying, has demonstrated the highest efficacy in *in vitro* and on farm studies, however, due to its potentially hazardous properties, it is recommended that it is applied by specialist contractors [20, 23] and is difficult to apply safely on pig farms unless there is a long empty period. Glutaraldehyde and QAC combinations have been shown to have good effectiveness, even in the presence of some residual organic matter [16, 18, 22]. The intervention buildings in the study described here were significantly less likely to be positive for *Salmonella* than the control buildings after C&D. Only one intervention building (farm 228C) had detectable residual *Salmonella* contamination after C&D. The reason for this apparent failure is uncertain, but monophasic *S*. Typhimurium, the serovar isolated post C&D, was also found in mice on this farm. It is therefore likely that rodents may have re-contaminated disinfected surfaces after disinfectant had dried [10, 26].

Some variations in the susceptibility of *Salmonella* strains to disinfectants have also been observed, even within the *S*. Typhimurium serogroup [37], but significant resistance is unlikely due to the multiple cellular targets that are impacted by individual disinfectants, and no *Salmonella* contamination was observed after C&D in the control building (previously contaminated with the same serovar) in this farm. Furthermore, variations in the proficiency of the contractors employed to carry out the C&D routine in intervention buildings was reported, and farm 228C was not cleaned and disinfected as effectively as the other farms. The proficiency of the operator carrying out the C&D routine is a critical factor, as the effective removal of organic matter, as well as accuracy in diluting and applying the disinfectant, can significantly impact on the removal of microorganisms [19, 20]. *Salmonella* contamination was observed post-C&D in the control buildings of four further farms. Three of these farms used a different glutaraldehyde and QAC product, but the dilution rate at which the disinfectant was applied (1:200) was far higher than the GO dilution (1:49). Over-diluting disinfectants is a common reason for disinfection failure [10, 23] and it is often related to lack of knowledge amongst farmers of the appropriate concentrations for disinfectants for bacterial pathogens [20]. The fourth control farm with residual post C&D contamination used an iodine-based disinfectant. In this farm, the disinfectant was used at a lower dilution rate (1:50) than the recommended GO rate (1:90). However, iodine-based compounds have been demonstrated to be less effective than aldehydes, especially in the presence of organic matter [18, 22]. This was confirmed in this study by the results of the multivariable analysis that showed that the iodine-based product used in farm 221C was significantly more likely to result in residual *Salmonella* contamination. The average post-C&D Enterobacteriaceae counts in this farm were amongst the highest in the study (5.32 log_10_CFU/50cm^2^), indicating significant residual faecal bacterial contamination, which could have inhibited the action of the disinfectant against *Salmonella*.

The majority of the *Salmonella-*positive samples after C&D were isolated from the floors of pens (12 from a total of 15, Table 3). This can be explained by the fact that concrete floors are rough surfaces that receive most faecal contamination when pens are occupied and are therefore more likely to have high levels of residual contamination [38]. Also, floor cracks are difficult to clean and can harbour residual contamination [17, 23]. The Enterobacteriaceae counts were significantly higher in intervention buildings. This was mainly due to high counts in the floors of farms 224C and 228C (data not shown) and may be related to the differential occurrence of specific disinfectant-tolerant bacteria amongst these pens, but this was not investigated further in this study. TBC counts were significantly higher in control buildings, but the difference(0.16 log_10_CFU/50cm^2^) was small. Limited efficacy of cleaning and disinfection in reducing counts of aerobic indicator bacteria in field conditions has been reported before [39]. The apparent discrepancy between the effect of the disinfection protocols on *Salmonella* and indicator organisms requires further investigation and confirmation, since such hygiene indicators are widely used and may not always be appropriate for assessing disinfectant activity against specific pathogens.

Enterobacteriaceae and TBC were significantly lower in feeders and drinkers in the intervention buildings. This can be due to the fact that smooth surfaces are easier to clean and disinfect [38]. These findings contrast with those of Mannion et al 2007, who reported a high level of contamination in feeders and drinkers, possibly explained by splashing of contaminated material on to these fixtures during cleaning. In our study, in the intervention buildings, these fixtures were power washed and disinfected, but cross contamination during washing was avoided. In a recent study, feeders and drinkers were reported to be more difficult to clean than floors in pig buildings [40]. In our study, in the intervention buildings trained contractors thoroughly washed and disinfected this equipment, and this could account for the difference observed.A variety of *Salmonella* serovars were isolated from the pigs sampled in this study. The serovars isolated in the five farms that had residual contamination post-C&D were isolated at the post-restocking visit in four farms. Carry-over of *Salmonella* after ineffective C&D is not uncommon [7, 10]. However, apparent carry-over of infection is also documented for farms that had no detectable residual contamination in the houses post C&D. This could be due to failing to detect low levels of residual contamination (for example when areas that are difficult to sample are contaminated, e.g. within feed pipes), but also could occur as a result of the presence of contaminated rodent populations, or via recontamination from residual material outside the pig pens that can be introduced by the movement of pigs or staff [23, 41]. On the other hand, a low level of residual contamination after C&D does not always lead to infection of the next group of animals, as reported for chicken flocks [20]. The majority of the *Salmonella* serovars isolated at the post-restocking visit were those also found at the pre-restocking visit. The pigs at the pre-restocking visit also had a significantly higher *Salmonella* prevalence. This could be due to the fact that the pigs sampled were young (4 weeks for all farms, apart from 224C and 230C where the pigs were sampled at 10 weeks of age), and therefore more likely to be shedding *Salmonella* at higher levels [9]. The prevalence of *Salmonella* shedding has been shown to be higher in weaner and grower pigs, and to decrease after 10 weeks of age [30]. *S*. Typhimurium or its monophasic variants were isolated from all farms. *S*. Typhimurium is commonly found in pigs [42], but in recent years the most common types of *Salmonella* isolated from pig farms in the UK are the monophasic *S*. Typhimurium variants [43]. All other *Salmonella* serovars isolated in this study are routinely found in pigs in the UK, some more commonly (e.g. *S*. Reading) and some infrequently such as *S*. Rissen [30, 44].

Whilst there was no difference in the *Salmonella* prevalence between intervention and control buildings at the post-restocking visit, a significant difference was observed at the pre-slaughter visit, where pigs housed in the intervention buildings had a significantly lower prevalence. This is consistent with the findings of [45] who reported that increased frequency and efficiency of cleaning reduces the prevalence of *S*. Typhimurium at slaughter, and emphasises the potential public health benefits of effective farm intervention measures [8].

Individual faecal samples were less likely to be positive for *Salmonella* than the pooled faecal samples. This is not unexpected as infected pigs shed *Salmonella* intermittently [12] and pooled faeces containing accumulated naturally mixed faecal material from droppings areas within pens are considered a sensitive measure of pen contamination [46].

Samples collected during the summer were more likely to be positive for *Salmonella*. However, when season was added to the multivariable model only the results from autumn were significantly associated with a higher odds of being posiitve once the intervention and effect of the other variable had been accounted for. The highest *Salmonella* prevalence is observed on farms in the summer months, and this can be attributed to the fact that the higher temperature represents a stress factor for the pigs and it can result in higher shedding rates [47].

The results obtained in the multivariable anlaysis might not be representative of all pig farms, as only 10 study farms were included in the model, and participation in this study was voluntary, but they provide indication of factors that can be aid the C&D process. The results of the multivariable analysis showed how thorough cleaning and disinfection of ledges, beams, vents and ceilings and allowing 3–10 days downtime between batches was an effective measure to reduce the likelihood of residual *Salmonella* contamination. Leaving pens empty for longer period (2–3 weeks) appeared to be a significant risk and this may reflect a less intensive management system on these farms. The time left for a pen to dry after cleaning was a risk factor with 3–4 days showing a higher risk than 1–2 days. However, this may have been a proxy for farms that used contractors rather than those that used their own staff. A roof ventilation system was a protective factor when compared to buildings with side vents, possibly because roof vents are easier to clean. The subjective cleanliness score given to each building by the sampling team at each visit indicated that buildings scored 2 (poor) were at greater risk than cleaner scores, and the use of straw as bedding was protective. Other significant risk factors included changing feed between visits, coughing present in the pigs, the use of treatments between visits, whereas improvements to wildlife control and harbourage was identified as a significant protective factor. These individual factors appeared to explain the difference between the results from the intervention and control buildings and may highlight the key differences between the cleaning protocols.

Rodent presence was detected by cameras at 9/10 farms and either house mouse or Norway rat carcases were obtained in all study farms. This is not unexpected as rodents are attracted to livestock farms by the presence of harbourage and feed [10, 48]. Premises with lower levels of harbourage tend to have lower levels of rat activity and it has previously been recommended that harbourage is kept to a minimum within 20m of the pig buildings, e.g. by using concrete or short mowed grass [48]. Harbourage availability and rodent population sizes were reduced after provision of advice by rodent specialists. Farm-specific audits and linked targeted advice has been shown to be a useful tool for encouraging improved control of *Salmonella* on positive farms [49, 50]. We were also able to make a preliminary comparison of activity indices from camera traps and trapping data for house mice; this gave a possible indication of the relationship between activity indices and population size, although further validation work is required to confirm this result.

*Salmonella* was isolated from a limited number of rodent carcasses (7.0% of those tested) and only from three study farms. These results are in agreement with a recent study conducted in Spain, where 10.2% of the rodent carcasses collected from 46.2% of the study farms were positive for *Salmonella* [29]. Previous studies performed in laying hen farms contaminated with *Salmonella* Enteritidis reported higher levels of infection in rodents [26]. It has been suggested that *S*. Enteritidis provokes a systemic infection in mice, whilst other serovars, such as *S*. Infantis, occur as intestinal carriage [51]. In experimentally infected mice, it has been shown that, even though all mice were successfully infected with *S*. Typhimurium, only 27.0% shed high levels of *S*. Typhimurium in their faeces, and that shedding was intermittent [52]. The *Salmonella* serotypes isolated in our study were the same as those circulating in pigs on the farm. It is therefore most likely that pigs represented the source of infection for the rodents, but that the rodents facilitated the persistence of *Salmonella* between batches of pigs [10, 29].

In conclusion, this study demonstrates that an appropriate disinfection programme aimed at eliminating *Salmonella* significantly reduces the likelihood of residual contamination of *Salmonella* positive pig buildings, and significantly reduces the prevalence of *Salmonella* prior to slaughter in the pigs from well cleaned and disinfected buildings. Due to the high prevalence of infection in replacement breeding and weaned pigs, elimination of *Salmonella* from pig holdings is unlikely to be possible in most countries. Rodents may play a role in the carry-over of infection of several pathogens between batches and should be effectively controlled. Cleaning and disinfection is a useful measure to reduce the proportion of infected pigs prior to slaughter, but is only one of many combinations of measures needed to minimise *Salmonella* contamination of pig meat.

## Supporting information

S1 File Complete dataset including sample level results and risk factors. (CSV)

## References

[1] EFSA. The European Union Summary Report on Trends and Sources of Zoonoses,Zoonotic Agents and Food-borne Outbreaks in 2014. EFSA Journal. 2015;13(12):4329. 10.2903/j.efsa.2018.5500 PMC7009540 32625785

[2] EFSA. Analysis of the baseline survey on the prevalence of *Salmonella* in holdings with breeding pigs in the EU, 2008. EFSA Journal. 2008;7(12):1377–470.

[3] EFSA. Report of the Task Force on Zoonoses Data Collection on the analysis of the baseline survey on the prevalence of *Salmonella* in slaughter pigs, in the EU, 2006–2007. EFSA Journal. 2008;135:1–111.

[4] Powell LF, Cheney TE, Williamson S, Guy E, Smith RP, Davies RH. A prevalence study of Salmonella spp., Yersinia spp., Toxoplasma gondii and porcine reproductive and respiratory syndrome virus in UK pigs at slaughter. Epidemiol Infect. 2016;144(7):1538–49. doi: 10.1017/S0950268815002794 26586451 10.1017/S0950268815002794 PMC9150582

[5] Bollaerts K, Messens W, Aerts M, Dewulf J, Maes D, Grijspeerdt K, et al Evaluation of scenarios for reducing human salmonellosis through household consumption of fresh minced pork meat. Risk analysis: an official publication of the Society for Risk Analysis. 2010;30(5):853–65. doi: 10.1111/j.1539-6924.2010.01368.x . 20199654 10.1111/j.1539-6924.2010.01368.x

[6] Berriman AD, Clancy D, Clough HE, Armstrong D, Christley RM. Effectiveness of simulated interventions in reducing the estimated prevalence of Salmonella in UK pig herds. PloS one. 2013;8(6):e66054 PubMed Central PMCID: PMC3695987. doi: 10.1371/journal.pone.0066054 23840399 10.1371/journal.pone.0066054 PMC3695987

[7] Beloeil PA, Chauvin C, Proux K, Fablet C, Madec F, Alioum A. Risk factors for Salmonella seroconversion of fattening pigs in farrow-to-finish herds. Veterinary research. 2007;38(6):835–48. doi: 10.1051/vetres:2007034 17903417 10.1051/vetres:2007034

[8] Bahnson PB, Fedorka-Cray PJ, Ladely SR, Mateus-Pinilla NE. Herd-level risk factors for Salmonella enterica subsp. enterica in U.S. market pigs. Preventive veterinary medicine. 2006;76(3–4):249–62. doi: 10.1016/j.prevetmed.2006.05.009 16828183 10.1016/j.prevetmed.2006.05.009

[9] De Busser EV, De Zutter L, Dewulf J, Houf K, Maes D. Salmonella control in live pigs and at slaughter. Veterinary journal. 2013;196(1):20–7. doi: 10.1016/j.tvjl.2013.01.002 23414643 10.1016/j.tvjl.2013.01.002

[10] Andres VM, Davies RH. Biosecurity measures to control *Salmonella* and other infectious agents in pig farms: a review. Comrehensive Reviews in Food Science and Food Safety. 2015 doi: 10.111/1541-4337,12137

[11] Fosse J, Seegers H, Magras C. Prevalence and risk factors for bacterial food-borne zoonotic hazards in slaughter pigs: a review. Zoonoses and public health. 2009;56(8):429–54. doi: 10.1111/j.1863-2378.2008.01185.x 19175574 10.1111/j.1863-2378.2008.01185.x PMC7165994

[12] Fedorka-Cray PJ, Whipp SC, Isaacson RE, Nord N, Lager K. Transmission of Salmonella typhimurium to swine. Veterinary microbiology. 1994;41(4):333–44. 7801533 10.1016/0378-1135(94)90029-9

[13] Beloeil PA, Fravalo P, Fablet C, Jolly JP, Eveno E, Hascoet Y, et al Risk factors for Salmonella enterica subsp. enterica shedding by market-age pigs in French farrow-to-finish herds. Preventive veterinary medicine. 2004;63(1–2):103–20. doi: 10.1016/j.prevetmed.2004.01.010 15099720 10.1016/j.prevetmed.2004.01.010

[14] Baloda SB, Christensen L, Trajcevska S. Persistence of a Salmonella enterica serovar typhimurium DT12 clone in a piggery and in agricultural soil amended with Salmonella-contaminated slurry. Applied and environmental microbiology. 2001;67(6):2859–62. PubMed Central PMCID: PMC92952. doi: 10.1128/AEM.67.6.2859-2862.2001 11375208 10.1128/AEM.67.6.2859-2862.2001 PMC92952

[15] Sandvang D, Jensen LB, Baggesen DL, Baloda SB. Persistence of a Salmonella enterica serotype typhimurium clone in Danish pig production units and farmhouse environment studied by pulsed field gel electrophoresis (PFGE). FEMS microbiology letters. 2000;187(1):21–5. 10828394 10.1111/j.1574-6968.2000.tb09130.x

[16] Gradel KO, Sayers AR, Davies RH. Surface disinfection tests with Salmonella and a putative indicator bacterium, mimicking worst-case scenarios in poultry houses. Poultry science. 2004;83(10):1636–43. 15510546 10.1093/ps/83.10.1636

[17] Luyckx KY, Van Weyenberg S, Dewulf J, Herman L, Zoons J, Vervaet E, et al On-farm comparisons of different cleaning protocols in broiler houses. Poultry science. 2015;94(8):1986–93. doi: 10.3382/ps/pev143 26047671 10.3382/ps/pev143

[18] Gosling RJ, Breslin M, Fenner J, Vaughan K, West E, Mawhinney I, et al An in-vitro investigation into the efficacy of disinfectants used in the duck industry against Salmonella. Avian pathology: journal of the WVPA. 2016:1–19. doi: 10.1080/03079457.2016.1188369 27207299 10.1080/03079457.2016.1188369

[19] Stringfellow K, Anderson P, Caldwell D, Lee J, Byrd J, McReynolds J, et al Evaluation of disinfectants commonly used by the commercial poultry industry under simulated field conditions. Poultry science. 2009;88:1151–5. doi: 10.3382/ps.2008-00455 19439623 10.3382/ps.2008-00455

[20] Carrique-Mas JJ, Marin C, Breslin M, McLaren I, Davies R. A comparison of the efficacy of cleaning and disinfection methods in eliminating Salmonella spp. from commercial egg laying houses. Avian Pathol. 2009;38:419–24. doi: 10.1080/03079450903193768 19937529 10.1080/03079450903193768

[21] Davies R, Breslin M. Observations on Salmonella contamination of commercial laying farms before and after cleaning and disinfection. The Veterinary record. 2003;152(10):283–7. 12650470 10.1136/vr.152.10.283

[22] McLaren I, Wales A, Breslin M, Davies R. Evaluation of commonly-used farm disinfectants in wet and dry models of Salmonella farm contamination. Avian pathology: journal of the WVPA. 2011;40(1):33–42. doi: 10.1080/03079457.2010.537303 . 21331946 10.1080/03079457.2010.537303

[23] Mueller-Doblies D, Carrique-Mas JJ, Sayers AR, Davies RH. A comparison of the efficacy of different disinfection methods in eliminating Salmonella contamination from turkey houses. Journal of applied microbiology. 2010;109(2):471–9. doi: 10.1111/j.1365-2672.2010.04667.x 20102426 10.1111/j.1365-2672.2010.04667.x

[24] Hancox LR, Le Bon M, Dodd CE, Mellits KH. Inclusion of detergent in a cleaning regime and effect on microbial load in livestock housing. The Veterinary record. 2013;173(7):167 PubMed Central PMCID: PMC3756521. doi: 10.1136/vr.101392 23839725 10.1136/vr.101392 PMC3756521

[25] Mannion C, Leonard FC, Lynch PB, Egan J. Efficacy of cleaning and disinfection on pig farms in Ireland. The Veterinary record. 2007;161(11):371–5. 17873266 10.1136/vr.161.11.371

[26] Davies RH, Wray C. Mice as carriers of Salmonella enteritidis on persistently infected poultry units. The Veterinary record. 1995;137(14):337–41. 8560683 10.1136/vr.137.14.337

[27] Henzler DJ, Opitz HM. The role of mice in the epizootiology of Salmonella enteritidis infection on chicken layer farms. Avian diseases. 1992;36(3):625–31. 1417592

[28] Umali DV, Lapuz RR, Suzuki T, Shirota K, Katoh H. Transmission and shedding patterns of Salmonella in naturally infected captive wild roof rats (Rattus rattus) from a Salmonella-contaminated layer farm. Avian diseases. 2012;56(2):288–94. doi: 10.1637/9911-090411-Reg.1 22856184 10.1637/9911-090411-Reg.1

[29] Andres-Barranco S, Vico JP, Garrido V, Samper S, Herrera-Leon S, de Frutos C, et al Role of wild bird and rodents in the epidemiology of subclinical salmonellosis in finishing pigs. Foodborne pathogens and disease. 2014;11(9):689–97. doi: 10.1089/fpd.2014.1755 24927384 10.1089/fpd.2014.1755

[30] Wales AD, McLaren IM, Bedford S, Carrique-Mas JJ, Cook AJ, Davies RH. Longitudinal survey of the occurrence of Salmonella in pigs and the environment of nucleus breeder and multiplier pig herds in England. The Veterinary record. 2009;165(22):648–57. 19946125 10.1136/vr.165.22.648

[31] Grimont P, Weill FX. Antigenic formulae of the Salmonella serovars. https://wwwpasteurfr/sites/wwwpasteurfr/files/wklm_enpdf. 2007.

[32] Bjornson B, Pratt H, Littig K. Control of domestic rats and mice Public health servic publication US Department of Health, Education and Welfare 1969;(563).

[33] Engeman RM. Indexing principles and a widely applicable paradigm for indexing animal populations. Wildlife Research. 2005;32(3):203–10.

[34] Lambert M, Callaby R, Budgey R, Bellamy F, Coats J, Talling J. Monitoring commensal rodernt populations on farms with camera traps: how not to fill your hard drive in six weeks. Pest Management Science 2016;Submitted.

[35] Bates D, Mächler M, Bolker B, Walker S. Fitting linear mixed-effects models using *lme4*. Journal of Statistical Software. 2015;67:1–48. doi: 10.18637/jss.v067.i01

[36] Rose N, Beaudeau F, Drouin P, Toux JY, Rose V, Colin P. Risk factors for Salmonella persistence after cleansing and disinfection in French broiler-chicken houses. Preventive veterinary medicine. 2000;44(1–2):9–20. 10727741 10.1016/s0167-5877(00)00100-8

[37] Thomson JR, Bell NA, M. R. Efficacy of some disinfectant compounds against porcine bacterial pathogens. Th Pig Journal. 2007;60:15–25.

[38] Madec F, Humbert F, Salvat G, Maris P. Measurement of the residual contamination of post-weaning facilities for pigs and related risk factors. Zentralblatt fur Veterinarmedizin Reihe B Journal of veterinary medicine Series B. 1999;46(1):37–45. 10085772 10.1046/j.1439-0450.1999.00207.x

[39] Luyckx K, Dewulf J, Van Weyenberg S, Herman L, Zoons J, Vervaet E, et al Comparison of sampling procedures and microbiological and non-microbiological parameters to evaluate cleaning and disinfection in broiler houses. Poultry science. 2015;94(4):740–9. doi: 10.3382/ps/pev019 25681611 10.3382/ps/pev019

[40] Luyckx K, Millet S, Van Weyenberg S, Herman L, Heyndrickx M, Dewulf J, et al Comparison of competitive exclusion with classical cleaning and disinfection on bacterial load in pig nursery units. BMC Veterinary Research. 2016;12(1):189 doi: 10.1186/s12917-016-0810-9 27600833 10.1186/s12917-016-0810-9 PMC5013629

[41] Martelli F, Gosling RJ, Callaby R, R. D. Observation on *Salmonella* contamination of commercial duck farms before and after cleaning and disinfection. Avian Pathol. 2016;In Press. doi: 10.1080/03079457.2016.1223835 10.1080/03079457.2016.1223835 27545288

[42] Mueller-Doblies D, Speed K, Davies RH. A retrospective analysis of Salmonella serovars isolated from pigs in Great Britain between 1994 and 2010. Preventive veterinary medicine. 2013;110(3–4):447–55. doi: 10.1016/j.prevetmed.2013.02.023 23561958 10.1016/j.prevetmed.2013.02.023

[43] Arnold ME, Gosling RJ, Martelli F, Mueller-Doblies D, Davies RH. Evaluation of the sensitivity of faecal sampling for detection of monophasic Salmonella Typhimurium and other Salmonella in cattle and pigs. Epidemiol Infect. 2015;143(8):1681–91. doi: 10.1017/S0950268814002453 25266772 10.1017/S0950268814002453 PMC9507225

[44] APHA. Salmonella in livestock production in GB in 2014. https://wwwgovuk/government/statistics/salmonella-in-livestock-production-in-great-britain-2014. 2015.

[45] Gautam R, Lahodny G Jr., Bani-Yaghoub M, Morley PS, Ivanek R. Understanding the role of cleaning in the control of Salmonella Typhimurium in grower-finisher pigs: a modelling approach. Epidemiol Infect. 2014;142(5):1034–49. doi: 10.1017/S0950268813001805 23920341 10.1017/S0950268813001805 PMC9151178

[46] Arnold ME, Cook A, Davies R. A modelling approach to estimate the sensitivity of pooled faecal samples for isolation of Salmonella in pigs. Journal of the Royal Society, Interface / the Royal Society. 2005;2(4):365–72. doi: 10.1098/rsif.2005.0057 ; PubMed Central PMCID: PMC1578272. 16849194 10.1098/rsif.2005.0057 PMC1578272

[47] Akil L, Ahmad HA, Reddy RS. Effects of climate change on Salmonella infections. Foodborne pathogens and disease. 2014;11(12):974–80. PubMed Central PMCID: PMC4346543. doi: 10.1089/fpd.2014.1802 25496072 10.1089/fpd.2014.1802 PMC4346543

[48] Lambert M, Quy R, Amith R, Cowan D. The effect of habitat management on home-range size and survival of rural Norway rat populations. Journal of Applied Ecology. 2008;45:1753–61.

[49] Carrique-Mas JJ, Breslin M, Snow L, McLaren I, Sayers AR, Davies RH. Persistence and clearance of different Salmonella serovars in buildings housing laying hens. Epidemiol Infect. 2009;137(6):837–46. Epub 2008/11/20. doi: 10.1017/S0950268808001568 19017427 10.1017/S0950268808001568

[50] Martelli F, Birch C, Davies RH. Observations on the distribution and control of Salmonella in commercial duck hatcheries in the UK. Avian pathology: journal of the WVPA. 2016;45(2):261–6. doi: 10.1080/03079457.2016.1146820 . 27100154 10.1080/03079457.2016.1146820

[51] Lapuz R, Tani H, Sasai K, Shirota K, Katoh H, Baba E. The role of roof rats (Rattus rattus) in the spread of Salmonella Enteritidis and S. Infantis contamination in layer farms in eastern Japan. Epidemiol Infect. 2008;136(9):1235–43. doi: 10.1017/S095026880700948X 17988423 10.1017/S095026880700948X PMC2870913

[52] Lawley TD, Bouley DM, Hoy YE, Gerke C, Relman DA, Monack DM. Host transmission of Salmonella enterica serovar Typhimurium is controlled by virulence factors and indigenous intestinal microbiota. Infect Immun. 2008;76(1):403–16. PubMed Central PMCID: PMC2223630. doi: 10.1128/IAI.01189-07 17967858 10.1128/IAI.01189-07 PMC2223630

